# Neural Stem/Progenitor Cell Transplantation in Parkinson’s Rodent Animals: A Meta-Analysis and Systematic Review

**DOI:** 10.1093/stcltm/szac006

**Published:** 2022-03-23

**Authors:** Yifeng Zheng, Jun Zhou, Yisai Wang, Fanfan Fan, Shengwen Liu, Yu Wang

**Affiliations:** Department of Neurosurgery, Tongji Hospital, Tongji Medical College, Huazhong University of Science and Technology, Wuhan, People’s Republic of China; Department of Neurosurgery, Tongji Hospital, Tongji Medical College, Huazhong University of Science and Technology, Wuhan, People’s Republic of China; Department of Electrical and Computer Engineering, Rutgers University-New Brunswick, New Jersey, USA; Department of Neurosurgery, Tongji Hospital, Tongji Medical College, Huazhong University of Science and Technology, Wuhan, People’s Republic of China; Department of Neurosurgery, Tongji Hospital, Tongji Medical College, Huazhong University of Science and Technology, Wuhan, People’s Republic of China; Department of Neurosurgery, Tongji Hospital, Tongji Medical College, Huazhong University of Science and Technology, Wuhan, People’s Republic of China

**Keywords:** neural stem/progenitor cells, Parkinson’s disease, cell transplantation, meta-analysis, rodent animals

## Abstract

The effects of neural stem/progenitor cells (NSPCs) have been extensively evaluated by multiple studies in animal models of Parkinson’s disease (PD), but the therapeutic efficacy was inconsistent. Here, we searched 4 databases (PubMed, Embase, Scopus, and Web of Science) and performed a meta-analysis to estimate the therapeutic effects of unmodified NSPCs on neurological deficits in rodent animal models of PD. Data on study quality score, behavioral outcomes (apomorphine or amphetamine-induced rotation and limb function), histological outcome (densitometry of TH^+^ staining in the SNpc), and cell therapy-related severe adverse events were extracted for meta-analysis and systematic review. Twenty-one studies with a median quality score of 6 (range from 4 to 9) in 11 were examined. Significant improvement was observed in the overall pooled standardized mean difference (SMD) between animals transplanted with NSPCs and with control medium (1.22 for apomorphine-induced rotation, *P* < .001; 1.50 for amphetamine-induced rotation, *P* < .001; 0.86 for limb function, *P* < .001; and –1.96 for the densitometry of TH+ staining, *P* < .001). Further subgroup analysis, animal gender, NSPCs source, NSPCs dosage, and pretreatment behavioral assessment were closely correlated with apomorphine-induced rotation and amphetamine-induced rotation. In conclusion, unmodified NSPCs therapy attenuated behavioral deficits and increased dopaminergic neurons in rodent PD models, supporting the consideration of early-stage clinical trial of NSPCs in patients with PD.

## Graphical Abstract

**Figure:**
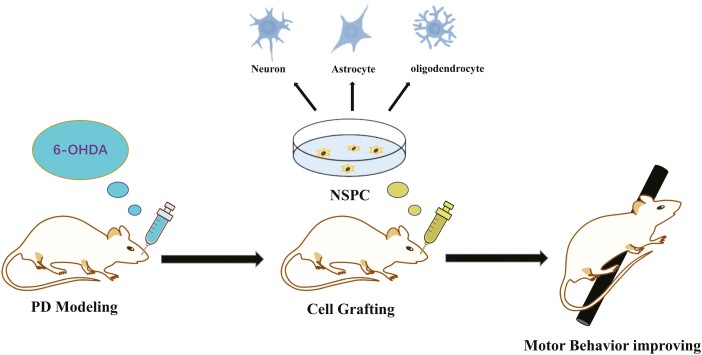
Neural stem progenitor cell (NSPC) is a cell stage capable of differentiation to the main phenotypes of the central nervous system (neuron and glial cells) in vitro and in vivo. After reviewing the investigations with unmodified NSPCs transplantation in rodent Parkinson’s animals, we systematically suggested a potential treatment effect of improving the behavioral motor deficits in Parkinson’s disease.

## Introduction

Parkinson’s disease (PD) is one of the most common chronic neurodegenerative diseases, with a reported standardized incidence rate being 8-18 per 100 000 person-year. In industrialized countries, the prevalence of PD is higher, at 0.3% of the entire population and approximately 1% in people over 60 years of age.^1-3^ Moreover, approximately 90% of cases are sporadic and have no identifiable genetic cause.^4^ Loss of dopamine-secreting neurons within the substantia nigra pars compacta (SNpc) and the presence of Lewy bodies are significant pathological findings in PD.^5^ The clinical manifestations of PD are diverse, including both motor and nonmotor features. At present, therapies are available for many nonmotor symptoms, including cholinesterase inhibitors for Parkinson’s disease dementia (PDD),^6^ antidepressants,^7^ and pramipexole^8^ for depression. However, motor symptoms, such as dysphagia, falls, and postural instability, tend to be treatment-resistant. Therefore, patients usually suffer from these motor disorders, which last for the rest of life and significantly reduce the life quality.^5^

There were many studies to explore the possibility of cell transplantation over 40 years ago. Among the varied cell types for PD cell therapy, neural stem/progenitor cells (NSPCs) and mesenchymal stem cells (MSCs) were both studied widely.^9,10^ Several published studies indicated that the functional recovery that occurs with MSCs transplanted therapy in animals is far more likely to cause by secreted biological factors that MSCs produce.^11,12^ In contrast, NSPCs were recognized as a more appropriate source owing to their capabilities of differentiation to the main phenotypes of the central nervous system in vitro and in vivo. Besides, they are also directly able to provide midbrain dopaminergic neurons, whether it derives from fetal tissue, embryonic stem cells (ESCs), or induced pluripotent stem cells (iPSCs).^10^ Multiple but inconsistent mechanisms about how NSPCs enhance functional recovery were proposed, such as neuroprotection and immunomodulation, and so forth.^13,14^ Similarly, the clinical curative benefit is conflicting among studies when the following factors are involved: cell source, state, dose, and treatment administration method.

The behavioral test allows insights into the functional benefits of a treatment. To consider the application of NSPCs in a clinical trial involving patients with PD, we performed a meta-analysis to review the preclinical studies and estimate the treatment effect of NSPCs on neurological deficits in rodent animal models of PD.

## Materials and Methods

### Search Strategy

Two independent investigators searched for correlative studies about NSPCs transplantation in rodent animal models of PD in 4 databases (PubMed, Embase, Scopus, and Web of Science databases, until September 10, 2021). The search strategy was as follows: ((neural stem cell) or (neural progenitor cell) or (neural precursor cell) or NSPC) and (Parkinson or PD or parkinsonian or PD). The default language for all included studies was English. We also searched the reference lists of eligible studies.

### Inclusion and Exclusion Criteria

According to the PICOS-scheme (population, intervention, control, outcome, and study design),^15^ the studies’ eligibility inclusion criteria were set up as follows: (1) PD model (rodent animals); (2) at least 1 experimental group tested the therapeutic effects of NSPCs; (3) sham-controlled (culture medium, or saline) group was set up; (4) providing adequate data on neurobehavioral function assessment or histological assessment; (5) original research studies; (6) published in English. The exclusion criteria were as follows: (1) studies that only evaluated the effects of transfected or modified NSPCs; (2) studies that only tested undifferentiated ESC, iPSC, or differentiated neuron precursor cells; (3) without precise animal numbers for individual comparison.

### Study Selection

After removing duplicates, all published articles were conducted by 2 investigators independently. Irrelevant studies were excluded with the agreement of investigators. All relevant articles were retrieved for a comprehensive review, and the criteria outlined above were used to evaluate the articles. Any controversies or uncertainties were judged by a third investigator and resolved through a 100% consensus and when necessary.

### Data Extraction

Two investigators abstracted the following information independently: (1) study characteristics (first author, year of publication); (2) features of the included animals (animal species, age, gender, numbers, PD model); (3) cell characteristics (cell source, administration time and site relative to lesion onset, and cell dosage); (4) follow-up period (administration of immunosuppressive drugs, the longest follow-up period of outcomes after NSPCs administration); (5) therapy-related adverse severe events (tumor/teratoma formation, infection, or death); (6) behavioral outcomes at the final time point recorded (amphetamine-induced rotation, apomorphine-induced rotation, and limb function); (7) histological outcome (densitometry of tyrosine hydroxylase-positive (TH^+^) staining in the SNpc). Limb function was defined as any test that analyzed forepaw use, such as the cylinder test, step test, and adhesive removal test. Get Data Graph Digitizer (version 2.24) was used to quantify the mean value and standard deviation (SD) or standard error (SE) from figures if only graphs were available. While only the standard error was reported, the standard deviation was converted by standard error with the following formula: SD=√N×SE, where *N* represents the size of the group. If research contained multiple experimental groups identified by different cell dosage or delivery sites, these groups would be included as independent studies respectively. Only the longest one was extracted when the outcomes were evaluated at different follow-up periods.

### Quality Assessment

Collaborative Approach to Meta-Analysis and Review of Animal Data from Experimental Studies (CAMARADES) checklist^16^ was used to estimate the methodological quality of all included studies, which consist of the following 11 items^17,18^: (1) publication in a peer-reviewed journal; (2) control of temperature; (3) random allocation to treatment or control; (4) allocation concealment; (5) blinded assessment of outcome; (6) avoidance of neuroprotective anesthetics (such as Ketamine); (7) animal model (aged, diabetic, or hypertensive); (8) sample size calculation; (9) compliance with animal welfare regulations; (10) statement of conflict of interest; (11) pretreatment behavioral assessment. One point for each item, with a total score of 11 points. Two investigators recorded a sum of the quality scores for each study independently. Any differences or uncertainty were resolved by consensus.

### Statistical Analysis

All statistical analyseswere performed with Stata (ver. 12.0, Stata Corp). The therapeutic effect size was calculated as standardized mean difference (SMD) by the random effect model and the statistic of Hedges.^19^ Overall, an effect size lesser than 0.2 represents a small effect, and an effect size greater than 0.8 is defined as a significant effect. Heterogeneity among studies is examined with Cochran’s Q-statistic test and represented by *I*^*2*^, and it was defined as low (25-50%), moderate (50%-75%), or considerable (>75%).^20^ A *P*-value of <.1) was considered statistically significant for heterogeneity.^21^ The statistical significance of the pooled effect size of all studies was performed by *t* test. A meta-regression analysis was performed while the heterogeneity was moderate or considerable according to several variables. Subgroup analysis with the following characteristics was used to investigate the possible relations with the neurological outcomes^22^: (1) Animal gender, (2) NSPCs source species (Allogeneic or Xenogeneic), (3) NSPCs state (pluripotent stem cell derivatives or primary cells), (4) NPSCs dosage (≤1E6, >1E6), (5) Administration time postinjury, (6) Administration site, (7) duration of follow-up period, (8) Design of pretreatment behavioral assessment. The interaction of the effects of different subgroups was tested based on random-effects models. Afterward, the potential publication bias was displayed using funnel plots, with an Egger test performed to evaluate the symmetry of the funnel plots.^23,24^

## Results

### Study Selection and Characteristics

**Figure 1.**
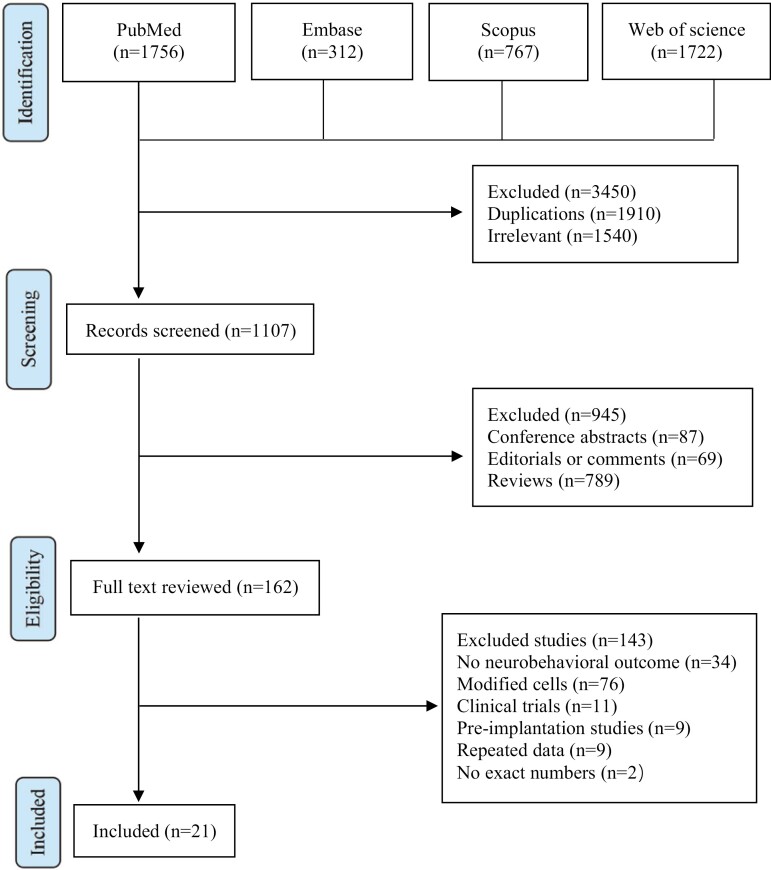
PRISMA flow diagram of included studies for this meta-analysis.

Figure 1 shows the search procedure and strategies. We searched a total of 2647 potential studies from 4 databases. After excluding 1540 irrelevant studies and 945 nonstandard research articles, 162 studies with full text were reviewed. According to the inclusion and exclusion criteria, 143 studies dissatisfying the eligibility criteria were excluded. Finally, a total of 21 studies without duplicate data descriptions were included in this meta-analysis.

The characteristic proportion of the 21 studies is summarized in Table 1 (see more details for each study in supplementary table 1). All parkinsonian models were from rodents and crated with 6-hydroxydopamine, a widespread PD model. Eleven studies (52.4%) used xenogeneic NPSCs as donors, most derived from humans. Meanwhile, 4 studies (19.0%) used NPSCs derived from pluripotent stem cells (ESC, MSC, or iPSC), while the others used primitive cells. The site of the NPSCs administration in most of the studies (71.4%) was located in the striatum of the brain. Only 6 (28.6%) studies chose other sites or combined with the striatum, such as the substantia nigra. Following 6-OHDA-induced injury, NPSCs were injected over a period varying from 1 to 7 weeks. The dosage of NPSCs ranged from 0.1 to 25 million cells per kilogram, with a median is 1.5 million. The median duration of the follow-up period on neurological assessment was 8 weeks. There were also 12 (57.2%) studies stating the usage of immunosuppression drugs and 9 (42.8%) studies without it.

**Table 1.** Characteristic proportion of 21 included studies.

Characteristics	Summary statistics
No. of publications, *n* (%)	21 (100%)
Animal species, *n* (%)	
Rat	20 (94.7%)
Mouse	1 (5.3%)
Animal gender, *n* (%)	
Male	12 (57.2%)
Female	7 (33.3%)
Unknown	2 (9.5%)
Lesion model, *n* (%)	
6-OHDA	21 (100%)
NSPCs source specie, *n* (%)	
Allogeneic	10 (47.6%)
Xonogeneic	11 (52.4%)
NSPCs state, *n* (%)	
PC-NSPC	17 (81.0%)
PSC-NSPC	4 (19.0%)
Administration time postinjury, *n* (%)	
≤2 week	5 (23.8%)
＞2 week	16 (76.2%)
Administration site, *n* (%)	
Striatum	15 (71.4%)
Substantia nigra	6 (28.6%)
Cell dosage (cells/kg), median (Q1, Q3)	1.5E+06 (5.0E+05, 2.5E+06)
Follow-up period (weeks), *n* (%)	
<12 weeks	12 (57.2%)
≥12 weeks	9 (42.8%)
Immunosuppressant, *n* (%)	
No	9 (42.8%)
Yes	12 (57.2%)
Behavioral outcome, *n* (%)	
Amphetamine-induced rotation	8 (38.1%)
Apomorphine-induced rotation	11 (52.4%)
Limb function	8 (38.1%)

Abbreviations: 6-OHDA, 6-hydroxydopamine; PC-NSPC, primary cells-neural stem/progenitor cells; PSC-NSPC, pluripotent stem cell-neural stem/progenitor cells; Q1, first quartile; Q3, third quartile.

For any study, if multiple behavioral tests were reported, we considered these different tests as independent experiments within one study. In general, 19 experiments were conducted to evaluate rotational behavior (amphetamine-induced rotation and apomorphine-induced rotation), and 8 experiments evaluated limb function (cylinder test, stepping test) (see Supplementary Table S3). About the histological outcome, the densitometry of TH+ staining in SNpc was assessed in 4 studies. Moreover, the survival rate of the grafted cells was observed in only 2 studies with no more than 5% successful survival rate after 6 weeks post-treatment (data not shown). For severe adverse events related to NSPCs transplantation, tumor formation was reported in 6 studies, and only 2 have reported animal deaths, with the exact number unknown. Considering that rotational behavior is the most common behavioral evaluation used in rodent PD studies, we took it as a primary outcome in this review.

### Quality Assessment

The median value of the quality score across all studies was 6, ranging from 3 to 8 (see more details in Supplementary Table S2). The distribution of the quality score meeting with each item is summarized in Table 2. In general, All the articles were published in a peer-reviewed journal, and no study used aged, diabetic, or hypertensive animals. Additionally, most studies claimed compliance with animal welfare regulations (89.5%), temperature control in the whole research (73.3%), as well as the behavioral training process before the treatment (78.9%). Although 11 studies randomly allocated the animals to the treatment or control group, only 4 studies used blinding when assessing the behavioral tests. However, neither the allocation concealment nor the Sample size calculation was performed by any of these studies.

**Table 2.** Distribution of the quality score meeting with each CAMARADES item.

Item	Number of studies	Percentage
Publication in a peer-reviewed journal	19	100.0%
Control of temperature	14	73.7%
Random allocation to treatment or control	11	57.9%
Allocation concealment	0	0.0%
Blinded assessment of outcome	4	21.1%
Avoidance of neuroprotective anesthetics	11	57.9%
Animal model (without aged, diabetic, or hypertensive)	19	100.0%
Sample size calculation	0	0.0%
Compliance with animal welfare regulations	17	89.5%
Statement of conflict of interest	12	63.2%
Pretreatment behavioral assessment	15	78.9%

### Effect Size

**Figure 2.**
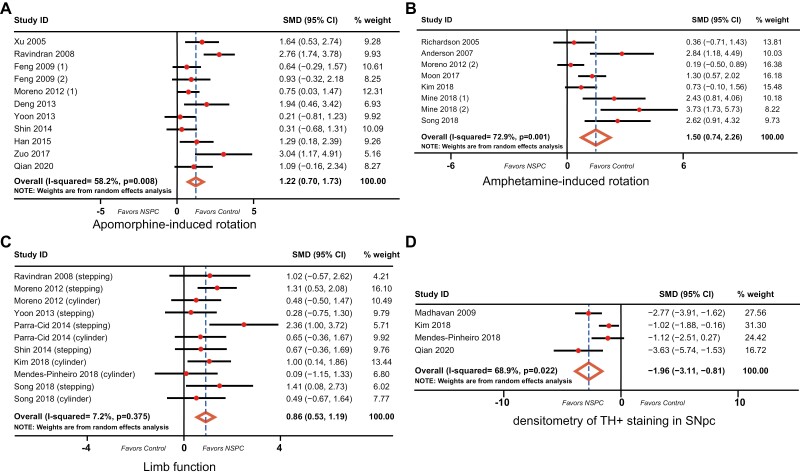
Forest plot of standardized mean difference (SMD) of 4 outcomes between NSPC therapy and control group along with a 95% confidence interval (95% CI). The degree of heterogeneity in the pooled estimates is represented at the I^2^ statistic. The overall estimate and confidence interval are marked by a diamond. NSPC, neural stem/progenitor cell.

The pooled effect size of NSPCs treatment was estimated based on the random-effects model and the Hedges calculation. Pooling the data of 11 studies that assessed the apomorphine-induced rotation (Fig. 2A) in PD animals showed a significant difference favoring NSPCs treatment (SMD: 1.22, 95% CI: 0.70-1.73, *P* <.001) with a moderate between-study heterogeneity (χ^2^ = 23.92, df = 10, *P = .*008, *I*^*2*^ =58.2%). Additionally, 8 studies assessed amphetamine-induced rotation (Fig. 2B) in 176 animals, and showed a significance difference in behavioral rotation favoring NSPCs group (SMD: 1.50, 95% CI: 0.74-2.26, *P* < .001) with a considerable between-study heterogeneity (*χ*^2^ = 25.85, df = 7, *P = .*001, *I*^*2*^ = 72.9%). Data on limb function are available in 11 studies. There is also a significant difference between the NSPCs group and control group (SMD: 0.86, 95% CI: 0.53-1.19, *P* < .001), with a low heterogeneity between studies (*χ*^2^ = 10.78, df = 10, *P = .*375, *I*^*2*^ = 7.2%; Fig. 2C); The densitometry of TH^+^ staining in SNpc was reported in 4 studies that investigated 71 animals. NSPCs were associated with a higher TH^+^ density in SNpc than was the control group. The difference is significant (SMD:‒1.96, 95% CI: –3.11 to 0.81, *P* < .001), with a considerable between-study heterogeneity (χ^2^ = 9.64, df = 3, *P = .*022, *I*^*2*^ = 68.9%; Fig. 2D).

### Subgroup Analysis and Meta-Regression Analysis

Following the effect size evaluation and sensitivity analysis, the between-study heterogeneity in apomorphine-induced rotation and amphetamine-induced rotation was still considerable. Therefore, subgroup analysis and meta-regression analysis based on 8 clinically related parameters were performed to investigate their contribution to the significant heterogeneity. Table 3 summarizes the data of apomorphine-induced rotation and amphetamine-induced rotation outcomes, respectively, in diverse subgroups. Generally, the significant therapeutic efficacy of NPSCs treatment was observed in most subgroups. However, some subgroups fail to reach the statistical significance (*P* < .05), which may be caused by insufficient studies. Additionally, moderate and considerable heterogeneity still could be detected in most subgroups (*I*^2^ > 50%). Although there was no significant between-study heterogeneity in several subgroups, we still could not identify the relevant clinical characteristic affecting the heterogeneity. To further investigate the unexplained between-study heterogeneity, univariate meta-regression analysis was used to test the influence of clinical characteristics. For apomorphine-induced rotation, the NPSCs state was the significant resource of the heterogeneity (Adj *R*^2^ = 75.90%, *P = .*058, < 0.01; Table 3). However, the assessment of pretreatment behavior was identified as a significant factor affecting the heterogeneity in amphetamine-induced rotation (*R*^2^ = 50.98%, *P = .*060, < .01; Table 3).

**Table 3.** Subgroup analysis and meta-regression of clinical variants correlated with apomorphine-induced rotation and amphetamine-induced rotation.

Clinical variants	Apomorphine-induced rotation								Amphetamine-induced rotation							
	No. of reports	Pooled estimates (SMD)	95% conf. Interval	*P*-value for SMD=0	*I* ^2^ value (%)	*P*-value for heterogeneity	*P*-value of interaction	Univariate analysis (Adj *R*2, *P*)	No. of reports	Pooled estimates (SMD)	95% conf. Interval	*P*-value for SMD=0	*I* ^2^ value (%)	*P*-value for heterogeneity	*P*-value of interaction	Univariate analysis (Adj R2, *P*)
Animal gender																
Male	6	1.432	(0.742, 2.121)	<.001	53.60%	.056	.610	–1.20%, 0.428	2	2.951	(1.689, 4.213)	<.001	0.00%	.324	.012	47.62%, .102
Female	3	1.083	(–0.069, 2.226)	.063	69.10%	.039			6	1.104	(0.389, 1.819)	.002	67.50%	.009		
NPSCs source species																
Allogeneic	5	1.148	(0.629, 1.667)	<.001	0.00%	.536	.810	–21.00%, 0.957	3	2.108	(0.007, 4.210)	.049	81.50%	.005	.459	–15.76%, .536
Xenogeneic	6	1.274	(0.402, 2.145)	.004	75.90%	.001			5	1.257	(0.438, 2.076)	.003	71.20%	.008		
NPSCs state																
PC-NSPCs	8	0.874	(0.398, 1.351)	<.001	32.90%	.166	.043	75.90%, 0.058	7	1.678	(0.771, 2.603)	<.001	76.10%	<.001	—	–18.08%, .479
PSC-NSPCs	3	1.918	(1.026, 2.809)	<.001	51.60%	.127			1	0.729	(–0.103, 1.560)	.086	NA	NA		
NPSCs dosage																
≤1E6	3	0.588	(–0.009, 1.185)	.054	0.00%	.743	.049	15.82%, 0.159	4	0.991	(0.216, 1.765)	.012	66.90%	.028	.174	7.49%, .266
>1E6	8	1.477	(0.820, 2.135)	<.001	63.30%	.008			4	2.215	(0.630, 3.799)	.006	76.20%	.006		
Administration time																
≤2 weeks	3	1.852	(0.798, 2.907)	.001	32.60%	.227	.187	3.63%, 0.233	1	0.359	(–0.711, 1.429)	.511	NA	NA	—	–4.92%, .325
>2 weeks	8	1.047	(0.476, 1.618)	<.001	61.60%	.011			7	1.703	(0.845, 2.561)	<.001	75.00%	.001		
Administration site																
Striatum	9	1.007	(0.547, 1.466)	<.001	37.40%	.120	.352	34.90%, 0.197	7	1.325	(0.562, 2.088)	.001	71.60%	.002	—	9.05%, .356
Substantia nigra	2	1.884	(0.094, 3.674)	.039	79.70%	.026			1	2.838	(1.185, 4.491)	.001	NA	NA		
Follow-up period																
<12 weeks	5	1.25	(0.312, 2.189)	.009	65.50%	.021	.968	–20.25%, 0.980	4	1.573	(0.297, 2.849)	.016	74.70%	.008	.952	–26.92%, .982
≥12 weeks	7	1.227	(0.579, 1.875)	<.001	58.80%	.033			4	1.523	(0.387, 2.659)	.009	78.50%	.003		
Pretreatment behavioral assessment																
Yes	8	1.439	(0.745, 2.134)	<.001	63.30%	.008	.119	2.96%, 0.276	6	2.002	(1.137, 2.868)	<.001	63.60%	.017	<.001	50.98%, .060
No	3	0.749	(0.233, 1.266)	.004	0.00%	.439			2	0.243	(–0.341, 0.826)	.415	0.00%	.800		

*I* ^*2*^ describes the variation in effect size attributable to heterogeneity. Adj *R*^2^ represents the proportion of between-study variance explained.

Abbreviations: APC-NSPC, primary cells-neural stem/progenitor cells; PSC-NSPC, pluripotent stem cell-neural stem/progenitor cells; SMD, standardized mean difference.

The interaction of diverse subgroups based on each characteristic was calculated with a random effect model. There is no comparative significance when a certain subgroup exists in one study. First of all, animal gender was correlated with the effect size of amphetamine-induced rotation (*P = .*012) but not on apomorphine-induced rotation (*P = .*610; Fig. 4A). NSPCs in male animals showed a greater efficacy. Allogenic and xenogeneic cells showed similar beneficial efficacy, although the former increased the amphetamine-induced rotation to a more considerable extent (Fig. 4B). But there was no significance (*P = .*459). All included studies did not provide a specific dosage of NSPCs. After standardized to animal weight, a more significant effect size with a higher dosage level (> 1 × 10^6^ cells/kg) on both apomorphine (*P = .*049) and amphetamine-induced rotation (*P = .*174) was observed (Fig. 4C). No significant correlation was found between administration time, administration site, and follow-up period. At last, the behavioral training before treatment was more beneficial to improve the effect size of both apomorphine (*P = .*019) and amphetamine-induced rotation (*P* < .001; Fig. 4D). Overall, since there were so many uncontrollable variables between different studies, the above various subgroup analysis can only generate hypotheses without confirming.

**Figure 3.**
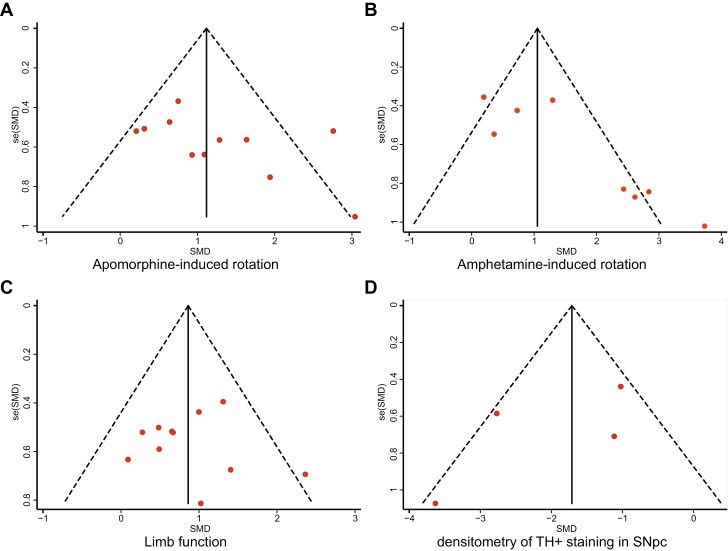
Funnel plot for apomorphine-induced rotation (A), amphetamine-induced rotation (B), limb function (C), and densitometry of TH^+^ staining (D). Individual study results are represented by dots, with the *y*-axis signifying study quality and the *x*-axis showing the study results. The solid vertical line represents the pooled effect size. The dashed diagonal lines represent pseudo-95% confidence limits around the pooled effect size for each standard error (SE) vertical axis. SMD, standardized mean difference.

**Figure 4.**
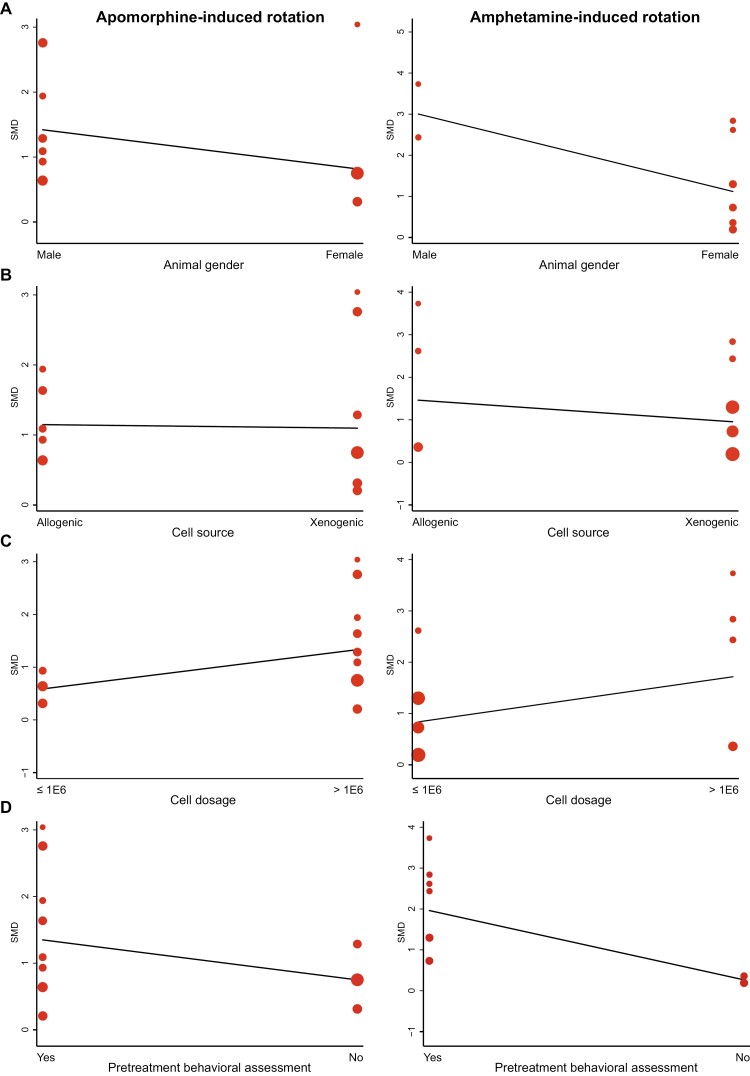
Meta-regression analysis for related variables and effect sizes of apomorphine-induced rotation and amphetamine-induced rotation. Individual study results are represented by dots, with the *y*-axis signifying the effect size. The size of the dot is determined by its weight. (A) Animal gender; (B) NPS cells source; (C) NPS cell dosage (cells/kg); (D) pretreatment behavioral assessment.

### Publication Bias

Figure 3 showes the funnel plots for the publication bias of apomorphine-induced rotation, amphetamine-induced rotation, limb function, and the densitometry of TH^+^ staining. No obvious publication bias was observed by visual observation. However, Egger’s test presented significant publication bias for amphetamine-induced rotation (*P = .*010). There was no publication bias detected for apomorphine-induced rotation (*P = .*117), limb function (*P = .*876), densitometry of TH^+^ staining (*P = .*350) by Egger’s test.

## Discussion

This meta-analysis revealed that treatment with NSPCs significantly improves neurological outcomes in rodent animal PD models. NSPCs treatment showed a significant effect size in either the behavioral deficit or pathology loss since the behavioral indicators could intuitively show the loss and recovery of motor function in animal PD models. At the same time, the densitometry of TH^+^ staining is publicly recognized as a dopaminergic neuron quantified approach. The extent of the 6-OHDA (an oxidative catecholaminergic toxin) lesion could easily be quantified according to the extent of apomorphine- or amphetamine-induced rotation,^25-27^ and this approach was widely used to evaluate the functional motor effects of dopaminergic neuron loss in the striatum. Recently, increasing evidence has suggested that limb function, another good indicator of nigrostriatal dopamine depletion, can also provide valuable clinically relevant data.^28,29^

Until now, studies about cell-based dopamine replacement strategies were initiated to explore the possibility of dopaminergic cell transplantation over 40 years ago. The existence of NSPCs has been known in developing or adult mammalian central nervous system (CNS) tissues, including humans.^30-32^ Fetal ventral mesencephalic (FVM) tissue grafting was performed to treat PD in the late 1970s.^33,34^ Although improvement was observed in multiple studies, including clinical trials,^35-37^ the results were not impressive enough. Moreover, there was growing concern about whether these foreign grafts are effective enough and whether they will cause further damage. Few studies observed that some patients developed severe graft-induced dyskinetic side effects postoperatively.^38,39^ Recently, ESCs and iPSCs have been widely researched as the most promising cell types because of their multi-directional differentiation potential.^40,41^ While transplantation of early-stage (undifferentiated) ESCs or iPSCs resulted in the spontaneous development of functional mDA neurons in vivo and significantly restored behavior in parkinsonian rats, it could cause inefficiency, inconsistent and incomplete mDA differentiation, and occasional teratoma formation.^41-45^ Furthermore, another critical question is whether those gene mutations negatively affect the survival and expansion of ESCs and iPSCs.^46,47^ To date, the origin of the epigenetic variability and its influence on the differentiation properties of iPSC lines remain controversial.^47^ In contrast, implantation of later stage, purified mDA neurons eliminated the formation of teratomas, but the transplanted neurons survived very poorly.^42-44^ Therefore, many investigators have turned to NSPCs, a “middle stage” that has the capacity for self-renewal and multipotent potential to become neurons or glial cells, which may represent a more optimal cell source than fully differentiated cells.^43,44,48^

As for the possible mechanism of NSPCs therapy on neuroprotective effect, it appears that multiple mechanisms may contribute, although it is still being elucidated. Unlike MSC therapy, NSPC treatment aims to specify neuron replacement, release specific neurotransmitters, and produce factors that promote neuronal growth and regeneration.^49^ In PD models, transplantation of NSPCs can replace dead and dying dopaminergic neurons due to the relatively focal nature of neuron loss that occurs. And transplanted-NSPCs have also been shown to establish synaptic connections with host neurons and release neurotransmitters or secretions. In addition, NSPCs can express a wide range of neurotrophic factors, such as BDNF, GDNF, and insulin-like growth factor 1 (IGF1), which demonstrate critical roles in the growth and stabilization of dendritic spines, synaptic plasticity, long-term potentiation, survival of neurons and glia, and therefore unsurprisingly motor performance.^50-52^

Although the effect size may be promising, several limitations still existed in our meta-analysis. First, with only 8 studies reported whether there were teratoma formation or animal death events and no detailed safety test on animals, it is inadequate to evaluate the clinical safety of NPSCs injection in animal PD models. Besides, our approach only covered those studies published in English. Unpublished data may change our results. Moreover, only 4 studies involved the test of the densitometry of TH+ staining in SNpc, indicating that more caution is needed in evaluating NSPCs therapy’s effect on rescuing dopaminergic neuron loss.

Although a considerable mean of the quality score (6.2) across all the studies in this meta-analysis was observed, some points are still worth paying attention to. Most low-quality studies (scoring less than 6.2) were published before 2012. These animal studies are commonly less rigorously designed, which may overestimate treatment effects and potentially influence results. A recognized and extensively applied CAMARADES list was used to evaluate the study quality. We believe that the items on this score list have an essential bearing on study quality. Although some items might be more important than others, and some may have been omitted, it is difficult to identify different items’ weight. The development of a more sophisticated quality score is a curial area for further research. Some items such as publication in a peer-reviewed journal, control of the temperature, randomization, blinded assessment of outcome, compliance with animal welfare regulations, and the statement of conflicts are widely accepted. For the rest, we believed that it is curial to induce blinded to allocation to prevent a bias in the severity of the induced injury. Besides, it is recognized that some anesthetics have much higher intrinsic neuroprotective activity, and their use is relevant to the study quality. Although sample size calculations are uncommon in animal studies, a good study should have an adequate sample size with a formal calculation.^53^ However, no studies in our meta-analysis performed sample size calculation, which suggested the lack of statistical power to ensure proper estimation of the treatment effects.^54^ Besides, the pretreatment behavioral training process could prevent a bias of the learning skill within the individuals. Their motor abilities would be utterly different without pretraining.

Moreover, the research models across all studies in this meta-analysis are based exclusively on healthy adult animals. However, the outcomes from rodent models cannot directly extend to humans, and their similarities to humans are limited. In addition, as PD is an age-related disease, a direct proportion between age and the prevalence of PD was stated.^55^ Moreover, clinically, the majority of PD patients are elderly. Therefore, it is necessary to consider the impact of age in preclinical studies, since the response to therapy may differ extensively in the developing, adult, and elderly brains. It is uncertain that cell therapy may not be able to obtain the same treatment effect in an elderly Parkinson’s animal model.

Another critical limitation in using a meta-analysis to assess potential therapeutic effects is publication bias. Although there is no significant publication bias for apomorphine-induced rotation, limb function, and the densitometry of TH^+^ staining, significant publication bias is existed in amphetamine-induced rotation, indicating that positive studies related to this behavioral test are more likely to be published compared with negative studies. Furthermore, motor tests in PD animal models do not adequately reflect all aspects of the disease’s neurological abnormalities. As a result of these limitations, our findings must be regarded with caution. Our findings should be validated in more strictly randomized control experiments and carefully interpreted in terms of the design of further animal investigations or clinical translation in the future, given the poor internal and external validity.

## Conclusion

This meta-analysis suggested a potential treatment effect of NSPCs over a wide range of doses, improving the behavioral function deficits in rodent animal models of PD, supporting the consideration of early-stage clinical trials of NSPCs in patients with PD.

## Supplementary Material

szac006_suppl_Supplementary_Material

## Data Availability

All data generated or analyzed during this study are included in this article.
