# Supplementary material for: Neural Stem/Progenitor Cell Transplantation in Parkinson’s Rodent Animals: A Meta-Analysis and Systematic Review
Source: Stem Cells Transl Med. 2022 Mar 23;11(4):383–93. doi: 10.1093/stcltm/szac006 (PMC9052406; doi:10.1093/stcltm/szac006)
Supplement: szac006_suppl_Supplementary_Material [file szac006_suppl_supplementary_material.pdf]

Table S1. Detailed Characteristics of 21 Reviewed Studies

Author	Year	Animal				PD model	Cell administration				Immuno suppressant	Follow-up period	Severe adverse event report
		Species	Age	Gender	Number		Source	Time (post-injury)	Site	Dosage (cells/Kg)			
Richardson[1]	2005	Fisher 344 rats	adult	male	14	6-OHDA	Adult rat brain	2 weeks	unilateral striatum	1.0E+06	no	2 weeks	-
Xu[2]	2005	Sprague-Dawley rats	adult	-	18	6-OHDA	Mouse ESC	4 weeks	unilateral striatum	2.5E+07	yes	6 weeks	Yes
Anderson[3]	2007	Lewis rats	adult	female	14	6-OHDA	Human fetal brain	3 weeks	substantia nigra	5.0E+05	yes	20 weeks	-
Ravindran[4]	2008	Wistar rats	adult	male	32	6-OHDA	Human ESC	4-6 weeks	substantia nigra	6.0E+06	yes	12 weeks	Yes
Madhavan[5]	2009	Fisher 344 rats	adult	male	25	6-OHDA	Rat fetal brain	1 week	substantia nigra	1.0E+06	no	5 weeks	-
Feng[6]	2009	Sprague-Dawley rats	adult	male	13	6-OHDA	Rat fetal brain	5 weeks	unilateral striatum	2.5E+05	no	16 weeks	-
Feng[6]	2009	Sprague-Dawley rats	adult	male	19	6-OHDA	Rat fetal brain	5 weeks	substantia nigra	2.5E+05	no	16 weeks	-
Moreno[7]	2012	Sprague-Dawley rats	adult	female	32	6-OHDA	Human NSC	6 weeks	unilateral striatum	1.5E+06	yes	16 weeks	-
Deng[8]	2013	Sprague-Dawley rats	adult	male	12	6-OHDA	Rat fetal brain	1 week	unilateral striatum	1.3E+06	no	8 weeks	-
Yoon[9]	2013	Sprague-Dawley rats	adult	-	15	6-OHDA	Human fetal brain	3 weeks	unilateral striatum	5.0E+06	Yes	8 weeks	-
Parra-Cid[10]	2014	Wistar rats	adult	male	16	6-OHDA	Mouse ENSC	7 weeks	substantia nigra	1.5E+06	No	3 weeks	-
Shin[11]	2014	Sprague-Dawley rats	adult	female	16	6-OHDA	Human NSC	4 weeks	unilateral striatum	4.0E+05	yes	8 weeks	-
Han[12]	2015	Sprague-Dawley rats	adult	male	16	6-OHDA	Human iPSC	4 weeks	unilateral striatum	2.5E+06	yes	16 weeks	-
Moon[13]	2017	Sprague-Dawley rats	adult	female	36	6-OHDA	Human fetal brain	4 weeks	unilateral striatum	2.3E+06	yes	12 weeks	Yes
Zuo[14]	2017	C57 mice	adult	female	12	6-OHDA	Human fetal brain	1 week	unilateral striatum	2.5E+06	no	8 weeks	-
Kim[15]	2018	Sprague-Dawley rats	adult	female	24	6-OHDA	Human MSC	4 weeks	unilateral striatum	1.5E+06	yes	9 weeks	Yes

MENDES-PINHEIR[16]	2018	Wistar rats	adult	male	10	6-OHDA	Human NSC	5 weeks	substantia nigra and striatum	2.0E+06	no	7 weeks	-
Mine[17]	2018	Sprague-Dawley rats	adult	male	22	6-OHDA	Swine fetal brain	4 weeks	unilateral striatum	1.0E+05	yes	8 weeks	Yes
Mine[17]	2018	Wistar rats	adult	male	22	6-OHDA	Rat fetal brain	4 weeks	unilateral striatum	1.0E+05	yes	8 weeks	Yes
Song[18]	2018	Sprague-Dawley rats	adult	female	12	6-OHDA	mouse fetal brain	4 weeks	unilateral striatum	4.5E+06	yes	24 weeks	-
Qian[19]	2020	Sprague-Dawley rats	adult	male	12	6-OHDA	Rat fetal brain	2 weeks	unilateral striatum	2.3E+06	no	12 weeks	-

These documents were ranked in order of the year. *NSC, neural stem cell; iPSC, induced pluripotent stem cell; ESC, embryonic stem cell; MSC, mesenchymal stem cell; -, not studeid.*

Supplementary References

1. Richardson, R.M., et al., *Grafts of adult subependymal zone neuronal progenitor cells rescue hemiparkinsonian behavioral decline.* Brain Res, 2005. **1032**(1-2): p. 11-22.
2. Xu, H., et al., *Neural precursor cells differentiated from mouse embryonic stem cells relieve symptomatic motor behavior in a rat model of Parkinson's disease.* Biochem Biophys Res Commun, 2005. **326**(1): p. 115-22.
3. Anderson, L. and M.A. Caldwell, *Human neural progenitor cell transplants into the subthalamic nucleus lead to functional recovery in a rat model of Parkinson's disease.* Neurobiol Dis, 2007. **27**(2): p. 133-40.
4. Geeta, R., et al., *One year survival and significant reversal of motor deficits in parkinsonian rats transplanted with hESC derived dopaminergic neurons.* Biochem Biophys Res Commun, 2008. **373**(2): p. 258-64.
5. Madhavan, L., et al., *Transplantation of subventricular zone neural precursors induces an endogenous precursor cell response in a rat model of Parkinson's disease.* J Comp Neurol, 2009. **515**(1): p. 102-15.
6. Zhu, Q., et al., *Grafted neural stem cells migrate to substantia nigra and improve behavior in Parkinsonian rats.* Neurosci Lett, 2009. **462**(3): p. 213-8.
7. Ramos-Moreno, T., C.G. Castillo, and A. Martinez-Serrano, *Long term behavioral effects of functional dopaminergic neurons generated from human neural stem cells in the rat 6-OH-DA Parkinson's disease model. Effects of the forced expression of BCL-X(L).* Behav Brain Res, 2012. **232**(1): p. 225-32.
8. Deng, X., et al., *Co-transplantation of GDNF-overexpressing neural stem cells and fetal dopaminergic neurons mitigates motor symptoms in a rat model of Parkinson's disease.* PLoS One, 2013. **8**(12): p. e80880.
9. Yoon, H.H., et al., *Are human dental papilla-derived stem cell and human brain-derived neural stem cell transplantations suitable for treatment of Parkinson's disease?* Neural Regen Res, 2013. **8**(13): p. 1190-200.
10. Parra-Cid, C., et al., *An enteric nervous system progenitor cell implant promotes a behavioral and neurochemical improvement in rats with a 6-OHDA-induced lesion.* Neurotoxicol Teratol, 2014. **43**: p. 45-50.
11. Shin, E.S., et al., *Enhanced efficacy of human brain-derived neural stem cells by transplantation of cell aggregates in a rat model of Parkinson's disease.* J Korean Neurosurg Soc, 2014. **56**(5): p. 383-9.
12. Han, F., et al., *Human induced pluripotent stem cell-derived neurons improve motor asymmetry in a 6-hydroxydopamine-induced rat model of Parkinson's disease.* Cytotherapy, 2015. **17**(5): p. 665-79.
13. Moon, J., et al., *Preclinical Analysis of Fetal Human Mesencephalic Neural Progenitor Cell Lines: Characterization and Safety In Vitro and In Vivo.* Stem Cells Transl Med, 2017. **6**(2): p. 576-588.
14. Zuo, F., et al., *Intrastriatal Transplantation of Human Neural Stem Cells Restores the Impaired Subventricular Zone in Parkinsonian Mice.* Stem Cells, 2017. **35**(6): p. 1519-1531.
15. Kim, H.W., et al., *Dual Effects of Human Placenta-Derived Neural Cells on Neuroprotection and the Inhibition of Neuroinflammation in a Rodent Model of Parkinson's Disease.* Cell Transplant, 2018. **27**(5): p. 814-830.
16. Mendes-Pinheiro, B., et al., *Secretome of Undifferentiated Neural Progenitor Cells Induces Histological and Motor Improvements in a Rat Model of Parkinson's Disease.* Stem Cells Transl Med, 2018. **7**(11): p. 829-838.
17. Mine, Y., et al., *Grafted Miniature-Swine Neural Stem Cells of Early Embryonic Mesencephalic Neuroepithelial Origin can Repair the Damaged Neural Circuitry of Parkinson's Disease Model Rats.* Neuroscience, 2018. **386**: p. 51-67.
18. Song, J.J., et al., *Cografting astrocytes improves cell therapeutic outcomes in a Parkinson's disease model.* J Clin Invest, 2018. **128**(1): p. 463-482.
19. Qian, Y., et al., *Transplantation of Nurr1-overexpressing neural stem cells and microglia for treating parkinsonian rats.* CNS Neurosci Ther, 2020. **26**(1): p. 55-65.

Table S2. CAMARADES checklists of All 21 reviewed studies

Study ID	Publication in a peer-reviewed journal	Control of temperature	Random allocation to treatment or control	Allocation concealment	Blinded assessment of outcome	Avoidance of neuroprotective anesthetics	Animal model (without aged, diabetic, or hypertensive)	Sample size calculation	Compliance with animal welfare regulations	Statement of conflict of interest	Pretreatment behavioral assessment	Score
Richardson 2005	yes	yes	yes	no	no	no	yes	no	yes	no	no	4
Xu 2005	yes	yes	no	no	no	yes	yes	no	yes	no	yes	5
Anderson 2007	yes	yes	no	no	no	yes	yes	no	no	yes	yes	5
Ravindran 2008	yes	no	no	no	yes	no	yes	no	yes	no	yes	5
Madhavan 2009	yes	yes	no	no	no	no	yes	no	yes	yes	no	5
Feng 2009	yes	no	no	no	yes	no	yes	no	no	no	yes	4
Moreno 2012	yes	yes	yes	no	no	no	yes	no	yes	no	yes	6
Deng 2013	yes	yes	yes	no	no	yes	yes	no	yes	no	yes	7
Yoon 2013	yes	yes	yes	no	no	yes	yes	no	yes	yes	yes	8
Parra-Cid 2014	yes	yes	yes	no	no	yes	yes	no	yes	yes	yes	8
Shin 2014	yes	no	no	no	no	yes	yes	no	yes	no	no	4
Han 2015	yes	no	no	no	no	yes	yes	no	yes	yes	no	5
Moon 2017	yes	yes	yes	no	no	no	yes	no	yes	yes	yes	7
Zuo 2017	yes	yes	yes	no	no	yes	yes	no	yes	yes	yes	8
Kim 2018	yes	yes	yes	no	no	no	yes	no	yes	yes	yes	7
Mendes-Pinheiro 2018	yes	yes	no	no	no	no	yes	no	yes	yes	yes	6
Mine 2018	yes	yes	yes	no	yes	yes	yes	no	yes	yes	yes	9
Song 2018	yes	yes	yes	no	yes	yes	yes	no	yes	yes	yes	9
Qian 2020	yes	no	yes	no	no	yes	yes	no	yes	yes	yes	7
**Total**	19	14	11	0	4	11	19	0	17	12	15	
**Percentage**	100.0%	73.7%	57.9%	0.0%	21.1%	57.9%	100.0%	0.0%	89.5%	63.2%	78.9%	

Table S3. Outcome type of all 21 included studies

Author	Year	Densitometry of TH+ staining in the SNpc	Apomorphine-induced rotation	Amphetamine-induced rotation	Limb function	
					Stepping test	Cylinder test
Richardson	2005	–	–	✓	–	–
Xu	2005	–	✓	–	–	–
Anderson	2007	–	–	✓	–	–
Ravindran	2008	–	✓	–	✓	–
Madhavan	2009	✓	–	–	–	–
Feng (1)	2009	–	✓	–	–	–
Feng (2)	2009	–	✓	–	–	–
Moreno	2012	–	✓	✓	✓	✓
Deng	2013	–	✓	–	–	–
Yoon	2013	–	✓	–	✓	–
Parra-Cid	2014	–	–	–	✓	✓
Shin	2014	–	✓	–	✓	–
Han	2015	–	✓	–	–	–
Moon	2017	–	–	✓	–	–
Zuo	2017	–	✓	–	–	–
Kim	2018	✓	–	✓	–	✓
Mendes-Pinheiro	2018	✓	–	–	–	✓
Mine (1)	2018	–	–	✓	–	–
Mine (2)	2018	–	–	✓	–	–
Song	2018	–	–	✓	✓	✓
Qian	2020	✓	✓	–	–	–
**Total**		4	11	8	6	5
